# Shotgun Metagenomic Sequencing Reveals Virome Composition of Mosquitoes from a Transition Ecosystem of North-Northeast Brazil

**DOI:** 10.3390/genes14071443

**Published:** 2023-07-14

**Authors:** Carine Fortes Aragão, Sandro Patroca da Silva, Bruna Laís Sena do Nascimento, Fábio Silva da Silva, Joaquim Pinto Nunes Neto, Valéria Cristina Soares Pinheiro, Ana Cecília Ribeiro Cruz

**Affiliations:** 1 Department of Arbovirology and Hemorrhagic Fevers, Evandro Chagas Institute, Secretariat of Health and Environment Surveillance, Ministry of Health, Ananindeua 67030-000, PA, Brazil; carinefaragao@gmail.com (C.F.A.); sandrosilva@iec.gov.br (S.P.d.S.); brunanascimento@iec.gov.br (B.L.S.d.N.); fabiosilva.analises@gmail.com (F.S.d.S.); joaquimneto@iec.gov.br (J.P.N.N.); 2 Laboratory of Medical Entomology, Stadual University of Maranhão, Caxias 65604-380, MA, Brazil; vc_pinheiro@hotmail.com

**Keywords:** metagenomic, metavirome, viruses, Culicidae, next-generation sequencing

## Abstract

A wide diversity of pathogenic mosquito-borne viruses circulate in the Brazilian Amazon, and the intense deforestation can contribute to the spread of these viruses. In this context, this study aimed to investigate the viral diversity in mosquitoes of the genera *Aedes*, *Culex*, *Haemagogus,* and *Sabethes* from a transition area between the Amazon, Cerrado, and Caatinga biomes in Brazil. Metagenomic high-throughput sequencing was used to characterize the virome of 20 mosquito pools. A total of 15 virus-like genomes were identified, comprising species genomically close to insect-specific viruses of the families *Iflaviridae*, *Metaviridae*, *Lispiviridae*, *Rhabdoviridae*, *Xinmoviridae*, and *Parvoviridae* and species of plant viruses of the families *Solemoviridae*, *Virgaviridae*, and *Partitiviridae*. However, sequences of viruses associated with human and animal diseases were not detected. Most of the recovered genomes were divergent from those previously described. These findings reveal that there are a large number of unknown viruses to be explored in the middle-north of Brazil.

## 1. Introduction

Mosquitoes (Diptera: Culicidae) are known to transmit a variety of viruses that cause diseases in humans and animals, such as Yellow fever, West Nile fever, and Japanese encephalitis [1]. For this reason, most research has focused on arboviruses for decades, with the maintenance of insect viromes unknown. In recent years, advances in next-generation sequencing (NGS) technologies and bioinformatics analysis have led to the discovery of a variety of novel viruses that infect insects themselves, the insect-specific viruses (ISVs) [2].

ISVs replicate exclusively in invertebrate cells and have no direct public or veterinary health impact [3,4]. However, numerous studies have shown that these viruses could interfere with the replication of some arboviruses in insect hosts, representing a biological strategy to reduce the occurrence of arboviruses [5,6,7]. In addition, some described ISVs are classified in the same viral families associated with arboviruses, such as *Flaviviridae*, *Rhabdoviridae*, *Togaviridae*, *Peribunyaviridae,* and *Phenuiviridae*, suggesting that these viruses may have an ancestral relationship [8].

In Brazil, a wide variety of wild-type arboviruses circulate, mainly in the Amazon region in the northern part of the country. More than 210 arboviruses have already been registered in this area [9], such as the *Ilheus* [10], *Rocio* [11], *Mucambo* [12], and *West Nile* [13] viruses. The wide diversity of vertebrate and invertebrate hosts and the tropical climate favor the maintenance of these viruses in this area [9]. However, the intense deforestation in the Amazon rainforest can be a crucial factor in arbovirus spread [14]. This context enhances the efforts for the realization of emerging viruses’ surveillance in transition areas with the Amazon region.

In this context, mosquitoes can be good environmental samplers due to their capacity to feed on a variety of vegetable (to obtain nectar) [15] and animal (for blood meal) hosts [16] allowing them to harbor a diversity of microorganisms that can habit the insect intestine transitorily or for long periods of time [17]. Therefore, metagenomic studies in mosquitoes can contribute to monitoring pathogenic viruses’ circulation and the discovery of novel virus species, elucidating insights about viral evolution. For example, an investigation based on metagenomic sequencing conducted in China identified arboviral (such as *Japanese encephalitis virus* and *Getah virus*) and ISV species in mosquitoes of the genera *Culex*, *Anopheles*, *Aedes,* and *Armigeres* [18].

In this study, we used a metagenomic-based approach to characterize the virome of wild-caught adult female mosquitoes of the genera *Aedes* (*Ae*.), *Culex* (*Cx*.), *Haemagogus* (*Hg*.), and *Sabethes* (*Sa*.) from three locations in a transition ecosystem between the North and Northeast of Brazil.

## 2. Materials and Methods

### 2.1. Collection Site

**Figure 1:**
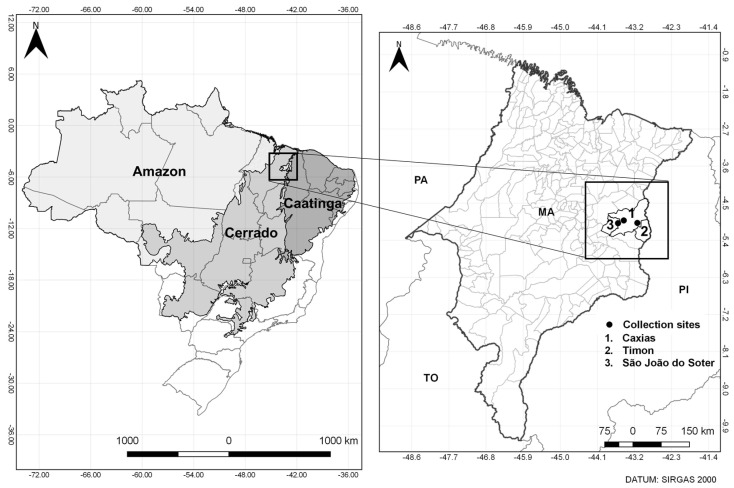
Map showing the mosquito collection sites in the municipalities of Caxias, Timon, and São João do Sóter, Maranhão State, Brazil. This area is situated in a transition ecosystem between Amazon, Cerrado, and Caatinga biomes. PA: Pará; MA: Maranhão; TO: Tocantins; PI: Piauí.

Collections were conducted in the proximities of Inhamum Municipal Environmental Protection Area (S 04°55′12.7″ W 043°27′37.0″), Pedras village (S 04°59′12.7″ W 043°35′59.1″), and Buriti Doce community (S 04°58′50.4″ W 043°07′51.2″), respectively located in the municipalities of Caxias, São João do Sóter, and proximities of Timon, located in Maranhão State, Brazil (Figure 1), in November 2021. Maranhão is in the Northeastern region of the country and is bounded by the Pará, Piauí, and Tocantins States. The three municipalities belong to the Cerrado biome and are situated in a transition area located between the Amazon and the semi-arid lands of the Brazilian northeast [19].

Areas with extensive vegetation cover and minimal anthropic action situated near the water sources were chosen to perform the collections. These activities were authorized by the Instituto Chico Mendes de Conservação da Biodiversidade (ICMBio) (grant number 68405-1).

### 2.2. Mosquito Collection and Taxonomic Identification

Adult mosquitoes were collected during six days using the Center of Disease Control’s (CDC) light traps installed at 1.50 m above ground level overnight and entomological nets using human attraction techniques for the collection of diurnal insects in the periods of 8 a.m. to 11 a.m. and 3 p.m. to 6 p.m. In all the collection sites, the mosquitoes were killed by dipping them in liquid nitrogen. The mosquitoes were morphologically identified to the most specific taxonomic level possible using species identification keys [20,21,22,23,24,25,26], quantified, and grouped in pools based on species, collection site, and date using 2 mL microtubes. Only females of the genera *Aedes*, *Culex*, *Haemagogus*, and *Sabethes* were selected for metagenomic analysis due to their being vectors of various viruses of medical and veterinary importance.

### 2.3. RNA Extraction, Library Preparation and Sequencing

Each pool of mosquitoes was homogenized in a microtube with a solution of 500 µL of Dulbecco’s phosphate buffered saline diluent 1X (D-PBS) (Life Technologies, Carlsbad, CA, USA) with 2% penicillin and streptomycin, 1% fungizone, and 5% fetal bovine serum and a 3 mm diameter tungsten sphere using TissueLyser II equipment (Qiagen, Hilden, Germany) at 25 Hz for 1 min based on an adapted protocol [27]. After the homogenized samples were centrifugated for 10 min at 10,000 rpm at 4 °C, 140 µL of the supernatant was collected for viral RNA extraction using the QIAamp viral RNA^®^ kit (Qiagen, Hilden, Germany), following the manufacturer’s guidelines.

The extracted RNA was used for synthesizing the first and second cDNA strands using SuperScript^TM^ VILO^TM^ MasterMix (Thermo Fischer Scientific, Waltham, MA, USA) and NEBNext mRNA Second Strand Synthesis Module (New England Biolabs, Ipswich, MA, USA) kits. Subsequently, cDNA libraries were constructed using the protocol for the SureSelect^QXT^ Whole Genome Library Prep (Agilent Technologies, Santa Clara, CA, USA) kit. Libraries were quantified by a Qubit^®^ 4.0 Fluorometer (Life Technologies, Waltham, MA, USA) and evaluated by an Agilent 2100 bioanalyzer (Agilent Technologies) for quality control. The libraries were sequenced by the paired-end method on the NextSeq 550 platform (Illumina, San Diego, CA, USA) using the NextSeq 500/550 High Output kit v.2.5 (300 cycles).

### 2.4. Bioinformatic and Phylogenetic Analysis

The raw sequence data were submitted to quality control analysis and treated to remove adapters and low-quality reads using fastp v0.23.2 [28], and to remove rRNA using SortMeRNA v4.3.6 (database smr_v4.3_default_db.fasta). De novo assembly was performed using SPAdes v3.15.5 (k-mers 21, 33, 55, and 77) and MEGAHIT v1.2.9 (k-mers 21, 31, 41, 51, 61, 71, 81, 91, and 99). The generated viral contigs were aligned against the non-redundant (nr) protein database by DIAMOND v2.0.15 [29] with a 10^−4^ e-value threshold and inspected with the MEGAN6 v6.21.1 program [30] to identify those corresponding to viral sequences. These contigs were explored using Geneious v.9.1.8 software. Domains were identified using InterProScan 5 (https://www.ebi.ac.uk/interpro/ accessed on 21 November 2022).

Nucleotide sequences of extracted contigs were compared with the sequences of the closest viral members available at the NCBI by multiple sequence alignment (MSA) using Clustal Omega to determine the phylogenetic relationships. A phylogenetic tree was constructed using the maximum likelihood (MV) method [31] in the IQ-TREE v.1.6.12 program, with the statistical support of an ultrafast bootstrap with 1000 replicates [32]. FigTree v.1.4.4 was used to visualize the phylogeny, and the final image was produced using InkScape v.1.1 software. The nucleotide and amino acid identities were obtained using Geneious v.9.1.8.

## 3. Results

### 3.1. Mosquito Collection and Generated Data

A total of 94 adult female mosquitoes of the genera *Aedes* (N = 49), *Culex* (N = 18), *Haemagogus* (N = 1), and *Sabethes* (N = 26) were collected and divided into 20 pools based on collection sites and species for high-throughput sequencing. Descriptions of each pool, including specie name, number of specimens, origination, and collection date, are shown in Table 1.

A total of 608,447,930 paired-end reads were generated by sequencing, of which 274,329,804 reads remained after quality control (Table 1).

### 3.2. Virome Analysis

Analyses of NGS viral reads identified 15 viral-like genomes, comprised of positive-sense single-stranded RNA (+ssRNA) viruses (families *Iflaviridae*, *Metaviridae*, *Solemoviridae*, and *Virgaviridae*), negative-sense ssRNA (−ssRNA) viruses (families *Lispiviridae*, *Rhabdoviridae*, and *Xinmoviridae*), double-stranded RNA (dsRNA) viruses (family *Partitiviridae*), and single-stranded DNA (ssDNA) viruses (family *Parvoviridae*) (Table 2).

#### 3.2.1. Iflaviridae

**Figure 2:**
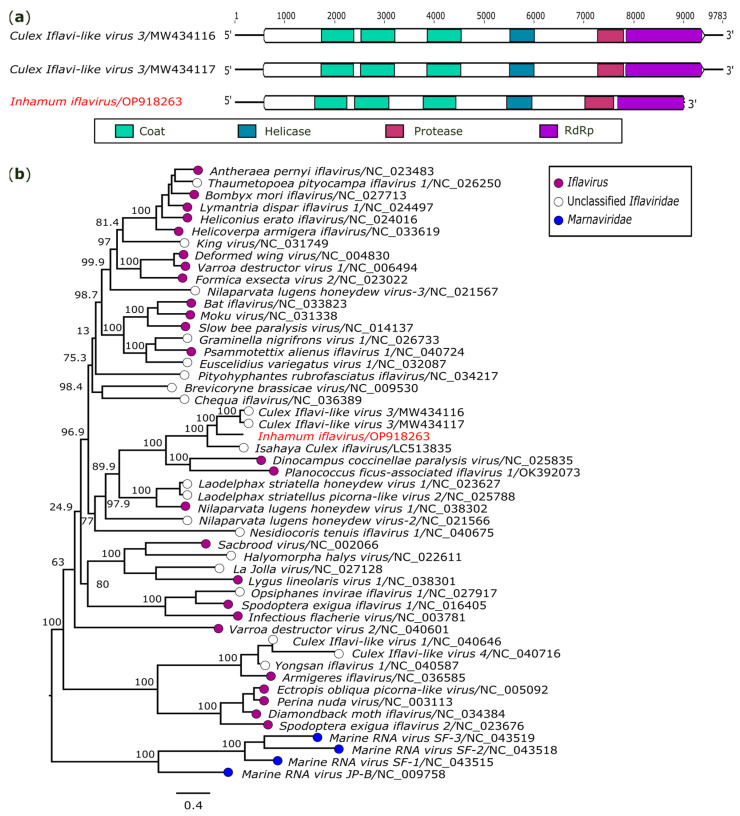
Genomic organization and phylogenetic relationship of *Inhamum iflavirus*. (**a**) Schematic representation of the obtained sequence compared with members of family *Iflaviridae*. Domains are displayed as colored boxes, and the sequence size is shown as number of nucleotides. (**b**) Maximum likelihood phylogenetic tree constructed based on the polyprotein nucleotide sequences. The bar corresponds to the nucleotide diversity along the branch of the tree. The virus identified in this study is indicated in red.

In the present investigation, a large genome of 9020 nucleotides (nt) in length closely related to members of the family *Iflaviridae* was obtained from a pool of *Cx.* (*Cux.*) spp. (AR872456). The open read frame (ORF), oriented from the 5′ to 3′ end and ranging from 585 to 9020 nt positions, encodes a polyprotein containing three structural proteins of the viral coat located at the N-terminal region and the non-structural proteins helicase, peptidase, and RdRp located at the C-terminal region (Figure 2a). This sequence has a similar structure and encodes the same proteins found in other iflavirures. However, the VPg at the 5′ terminus and the polyadenylated region at the 3′ terminus reported in iflaviruses were not recovered in the analysis.

The obtained polyprotein displayed the most significant identity with *Culex Iflavi-like virus 3* (GenBank accession: MW434116), presenting 59.9% (nt) and 58.2% (aa) of identity. Furthermore, the obtained sequence’s identity ranged from 7.6% (with *Culex Iflavi-like virus 4*/NC_040716) to 64.7% (with *Culex Iflavi-like virus 3*/MW434116) by comparing their three capsid protein domains with the same region of other members of the family *Iflaviridae*. According to the International Committee on Taxonomy of Viruses (ICTV), amino acid sequence identity in the capsid protein below 90% is one of the criteria used to define new iflaviruses [33]. Therefore, our data suggest the discovery of a novel iflavirus, tentatively named *Inhamum iflavirus* (IhIV) (GenBank accession: OP918263). The polyprotein nucleotide sequence of IhIV was aligned with 46 sequences of the family *Iflaviridae* and four other sequences of the family *Marnaviridae* that represented the external group. Phylogenetic analysis provides evidence of common ancestry shared between IhIV and iflaviruses (Figure 2b).

#### 3.2.2. Metaviridae

**Figure 3:**
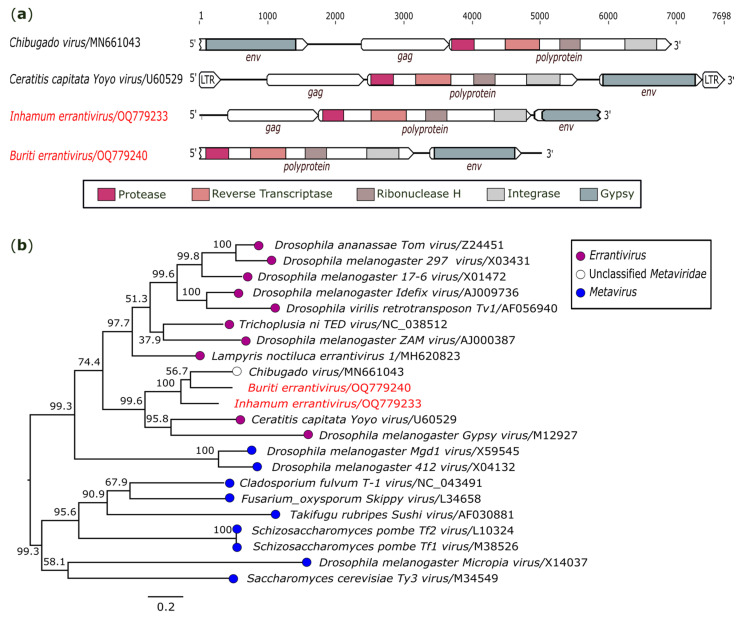
Genomic organization and phylogenetic relationship of *Buriti errantivirus* and *Inhamum errantivirus*. (**a**) Schematic representation of the obtained sequences compared with members of family *Metaviridae*. Domains are displayed as colored boxes, and the sequence size is shown as number of nucleotides. (**b**) Maximum likelihood phylogenetic tree constructed based on nucleotide sequences of the polyprotein region. The bar corresponds to the nucleotide diversity along the branch of the tree. The viruses identified in this study are indicated in red.

Two sequences closely related to members of the genus *Errantivirus* (family *Metaviridae*) were identified. The first sequence was obtained from *Sa. Chloropterus* (AR872465), and it contains 5823 nt in length. Oriented to the 5′ to 3′ terminus, the contig has three ORFs that encode: a putative gag protein (1332 nt), responsible for encoding the domains for the capsid and the nucleocapsid; a polyprotein (3120 nt) that includes domains for the typical protease (PR), reverse transcriptase (RT) domain, integrase (INT), and ribonuclease H (RH) enzymes found in metaviruses; and an incomplete ORF encoding the gypsy protein, associated with the products of the envelope gene (Figure 3a). This genome was closer to *Chibugado virus* (GenBank accession: MN661043), an unclassified *Metaviridae*, presenting 62.8% (nt) and 63.7% (aa) of identity regarding the conserved region, which contained PR, RT, RH, and INT proteins. The low identity suggests that we recovered a novel species, tentatively named *Inhamum errantivirus* (GenBank accession: OQ779233).

A second sequence was recovered from *Cx. usquatus* (AR872498). This sequence (5014 nt) has two ORFs. The first, located in the 5′ terminus, codifies a polyprotein that contains PR, RT, RH, and INT, whereas the second, located in the 3′ terminus, codifies the gypsy protein (Figure 3a). This genome shares 62.3% nt and 63.0% aa identity with *Chibugado virus* and is considered a novel species tentatively named *Buriti errantivirus* (GenBank accession: OQ779240). In addition, nucleotide and amino acid alignments showed that *Inhamum errantivirus* and *Buriti errantivirus* are divergent from each other, sharing 66.6% (nt) and 69.9% (aa) of identity based on the polyprotein region.

Despite the close identity shared between them, the genomic organization of *Chibugado virus* differs from other members classified in the family *Metaviridae*. In the 5’ to 3’ direction, *Chibugado virus* presents a genomic organization of env-gag-polyprotein, while the two sequences obtained here present the typical organization (gag-polyprotein-env) found in other members of the family (Figure 3a). A phylogenetic tree based on the polyprotein region showed that *Inhamum errantivirus* and *Buriti errantivirus* share ancestral relationships with other metaviruses (Figure 3b).

#### 3.2.3. Solemoviridae

**Figure 4:**
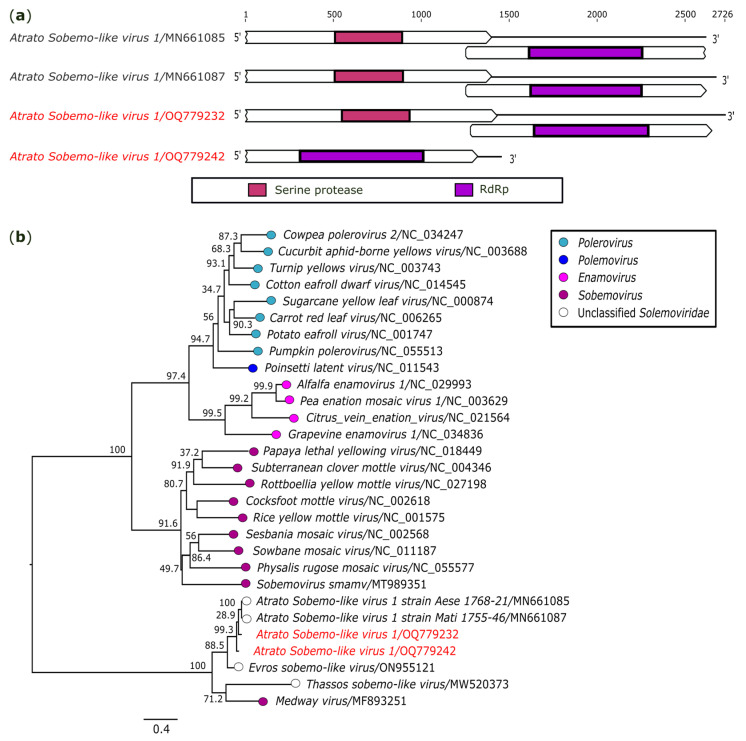
Genomic organization and phylogenetic relationship of the two strains of *Atrato Sobemo-like virus 1* identified in the present study (indicated in red). (**a**) Schematic representation of the obtained sequences compared with sequences corresponding to segment 1 of two strains ofASLV1. Domains are displayed as colored boxes, and the sequence size is shown as number of nucleotides. (**b**) Maximum likelihood phylogenetic tree constructed based on nucleotide sequences of *RdRp* gene. The bar corresponds to the nucleotide diversity along the branch of the tree.

In this study, genomes closely related to the unclassified solemovirus *Atrato Sobemo-like virus 1* strain Mati 1755-46 (ASLV1; GenBank accession: MN661087) were found in two pools (AR872461 and AR872510) of *Ae. Serratus.* The first is a genome of 2726 nt in length, composed of two ORFs that codify a peptidase protein and a RdRp domain (Figure 4a). The RdRp domain shares 93.3% (nt) and 97.1% (aa) of its identity with ASLV1.

Another sequence of 1451 nt corresponding to the RdRp region (Figure 4a) shares 92.3% (nt) and 98.3% (aa) of identity with ASLV1. Unfortunately, the typically 3′proximal ORFs that encode the coat protein found in solemoviruses [34] were not obtained in the two described genomes. The high identity indicates that the genomes recovered correspond to the same reference virus, ASLV1, and they were deposited on GenBank under the accession numbers OQ779232 and OQ779242.

A phylogeny based on the RdRp region placed the two sequences obtained in this study in a clade with unclassified members of the family *Solemoviridae* and a representative member of the genus *Sobemovirus* (*Medway virus*) (Figure 4b).

#### 3.2.4. Virgaviridae

**Figure 5:**
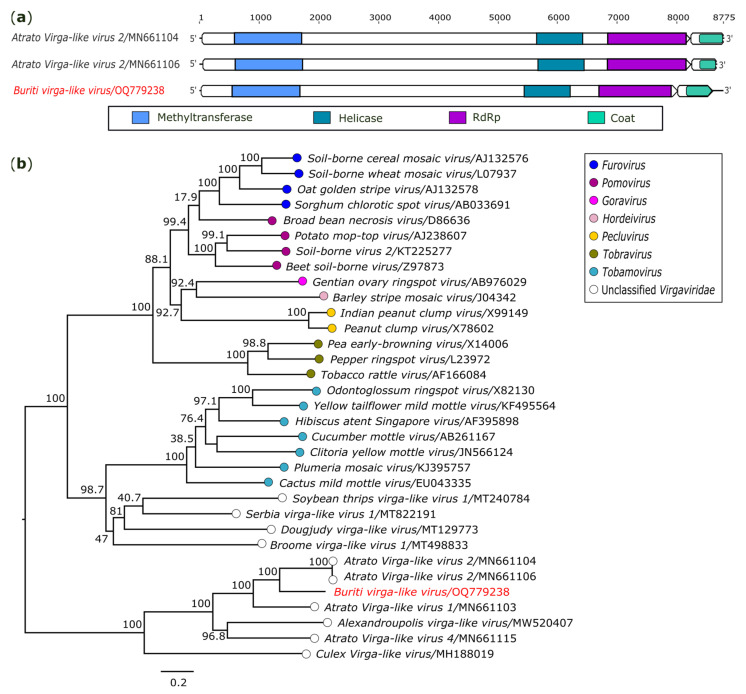
Genomic organization and phylogenetic relationship of *Buriti virga-like virus*. (**a**) Schematic representation of the obtained sequence compared with members of family *Virgaviridae*. Domains are displayed as colored boxes, and the sequence size is shown as number of nucleotides. (**b**) Maximum likelihood phylogenetic tree constructed based on nucleotide sequences of polyprotein region. The bar corresponds to the nucleotide diversity along the branch of the tree. The virus obtained in this investigation is shown in red.

In the present investigation, a genome with 8757 nt related to the family *Virgaviridae* was identified in *Sa. chloropterus* (AR872486). The largest ORF (7974 nt) located in the 5′ end encodes a polyprotein predicted in the methyltransferase, helicase, and RdRp domains. A short ORF (585 nt) present in the 3′ terminus codifies capsid protein (Figure 5a). The genome is closely related to *Atrato Virga-like virus 2* (GenBank accession numbers: MN661104 and MN661105) and presents 61.2% (nt) and 59.7% (aa) of identity for both strains, suggesting that it is a novel virus, tentatively named *Buriti Virga-like virus* (GenBank accession number: OQ779238). The putative novel virus clustered in a distinct clade with unclassified virgaviruses based on the nucleotide sequence alignment of the polyprotein (Figure 5b).

#### 3.2.5. Mononegavirales: Rhabdoviridae, Lispiviridae and Xinmoviridae

**Figure 6:**
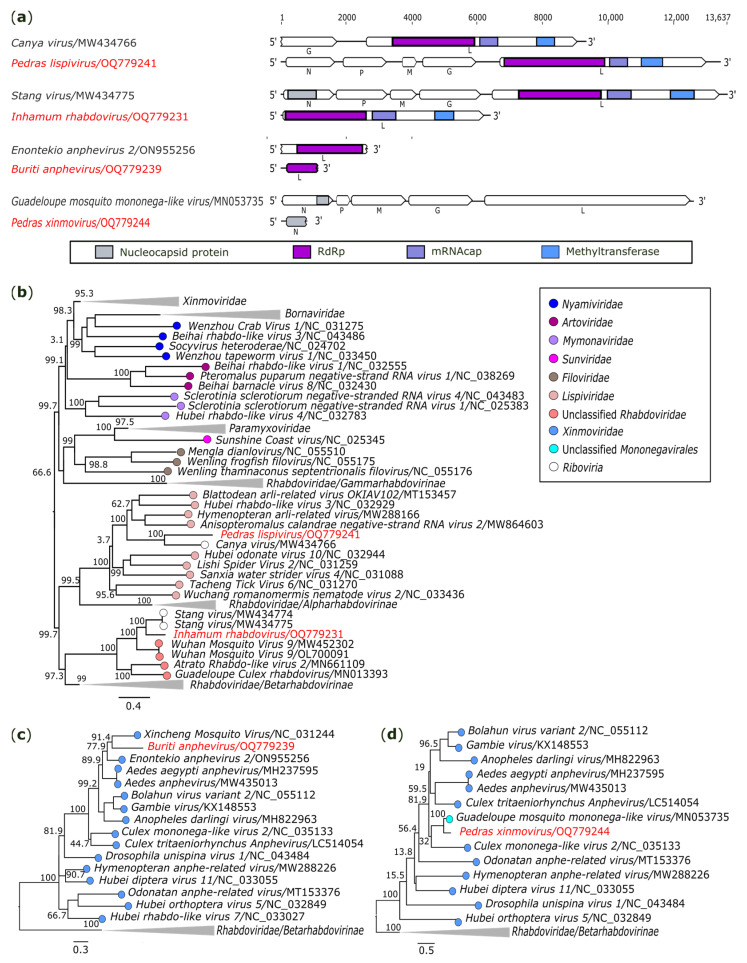
Genomic organization and phylogenetic relationship of the *Pedras lispivirus*, *Inhamum rhabdovirus*, *Buriti anphevirus*, and *Pedras xinmovirus*. (**a**) Schematic representation of the obtained sequences compared with members of order *Mononegavirales*. Domains are displayed as colored boxes, and the sequence size is shown as number of nucleotides. Genes *N*, *P*, *M*, and *G* of *Pedras lispivirus* were determined based on the closest hit in the BlastX search. Maximum phylogenetic trees were constructed based on the (**b**) polyprotein, (**c**) RdRp, and (**d**) nucleoprotein regions. The bar corresponds to the nucleotide diversity along the branch of the tree. The viruses obtained in this study are shown in red. N: nucleoprotein; P: phosphoprotein; M: matrix protein; G: glycoprotein; L: large (polymerase) domain.

In another analysis, two contigs showed near correspondence with unclassified *Riboviria* species. The first was obtained from *Sa. quasicyaneus* (AR872508), and it is a sequence of 13,424 nt, close to *Canya virus* (GenBank accesion: MW434766), sharing 46.1% (nt) and 35.5% (aa) of identity. The sequence contains five ORFs, presenting a similar genome organization found in mononegaviruses in the following order of genes oriented to the 5′ to 3′ terminus: *nucleoprotein* (N; 1470 nt), *phosphoprotein* (P; 1326 nt), *matrix protein* (M; 426 nt), *glycoprotein* (G; 1641 nt), and *polyprotein* (polymerase) (L; 6300 nt) (Figure 6a). In addition, Interproscan analysis identified *Mononegavirales*-specific domains, RdRp (IPR014023), mRNAcap (IPR026890), and methyltransferase (IPR039530), in the *L* gene, while the *N*, *P*, *M*, and *G* genes were determined based on the closest hit in the BlastX search (Figure 6a). The nucleotide sequences corresponding to the polyproteins of different mononegaviruses were used to determine phylogenetic relationships. The analysis indicates that the putative novel virus, tentatively named *Pedras lispivirus* (GenBank accession: OQ779241), belongs to the family *Lispiviridae* (Figure 6b).

**Figure 7:**
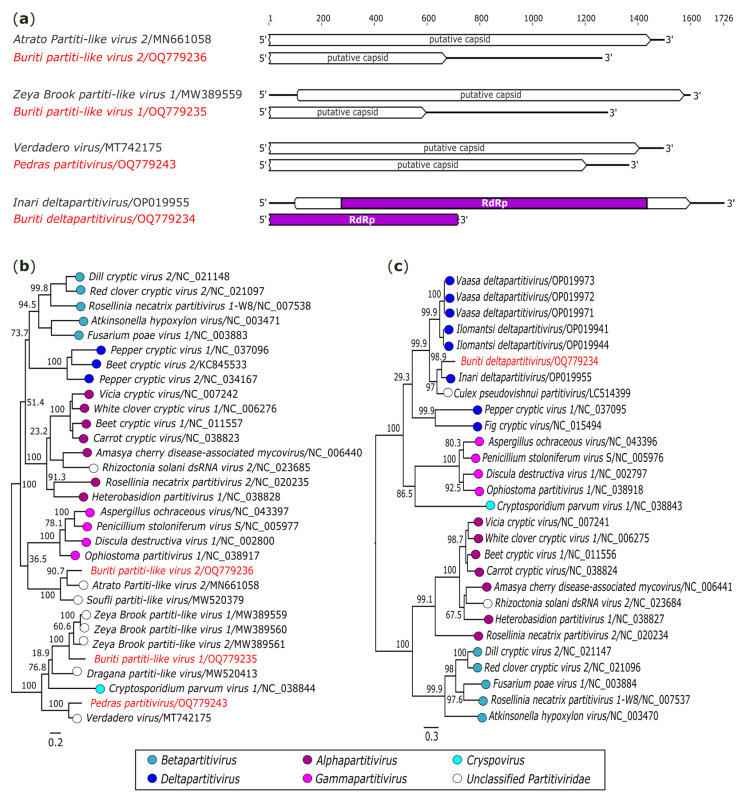
Genomic organization and phylogenetic relationship of *Buriti partiti-like virus 1*, *Buriti partiti-like virus 2*, *Pedras partitivirus*, and *Buriti deltapartitivirus*. (**a**) Schematic representation of the obtained sequences compared with members of family *Partitiviridae*. Domains are displayed as colored boxes. ORFs lacking domains were determined based on the closest hit in the BLASTX search. Sequence size is shown as number of nucleotides. Maximum likelihood phylogenetic trees constructed based on nucleotide sequences of (**b**) capsid protein and (**c**) *RdRp* genes. The bar corresponds to the nucleotide diversity along the branch of the tree. The viruses identified in this study are indicated in red.

The other contig closely associated with *Riboviria* sp. has 6397 nt, enclosing a single ORF, and was obtained from a pool of *Cx*. (*Cux*.) spp. (AR872456). It shares a closer similarity with the *Stang virus* (GenBank accession: MW434775), sharing 68.4% (nt) and 77.3% (aa) of identity. Just as was carried out for the previous genome, analysis of Interproscan identified the typical domains of mononegaviruses in the *L* gene (Figure 6a). Phylogenetic analysis of the obtained sequence with other mononegaviruses assembled it into a clade of unclassified *Rhabdoviridae* (Figure 7b). This putative novel virus was tentatively named *Inhamum rhabdovirus* (GenBank accession: OQ779231).

Two other sequences found were associated with the family *Xinmoviridae* (also belonging to the order *Mononegavirales*). A short and incomplete sequence of 1139 nt codifying a RdRp domain (Figure 6a) was identified in *Sa. glaucodaemon* (AR872487) and presented close nucleotide (56.0%) and amino acid (52.4%) identity with *Enontekio anphevirus 2* (GenBank accession: ON955256), belonging to the genus *Anphevirus* (*Xinmoviridae*). A phylogeny based on the RdRp showed that the recovered sequence has a relationship with other anpheviruses (Figure 6c). This supposed novel virus was tentatively named *Buriti anphevirus* (GenBank accession: OQ779239).

Another short sequence of 787 nt in length was found in *Ae. scapularis* (AR872521). The sequence corresponds to the nucleoprotein region (Figure 6a), and it was related to *Guadeloupe mosquito mononega-like virus* (GenBank accession: MN053735), an unclassified xinmovirus, with nucleotide and amino acid identities of 74.9% and 87.2%, respectively. Phylogenetic analysis showed that the recovered sequence clustered with other xinmoviruses (Figure 6d); however, the short fragment and the absence of conserved regions, such as the RdRp, preclude further inferences. This genome was tentatively named *Pedras xinmovirus* (GenBank accession: OQ779244).

#### 3.2.6. Partitiviridae

In the present investigation, three sequences presented correspondence to the capsid proteins of different partitiviruses (Figure 7a). A sequence of 1364 nt identified in *Ae. albopictus* (AR872511) had close relation to *Verdadero virus* (GenBank accession: MT742175), an unclassified virus of this family, with 71.4% (nt) and 80.0% (aa) of identity. The phylogenetic analysis showed that the recovered sequence clustered next to a member of the genus *Cryspovirus* and other unclassified partitiviruses (Figure 7b). This putative viral genome was named *Pedras partitivirus* (GenBank accession: OQ779243).

The two other sequences were recovered in a pool of *Sa. Quasicyaneus* (AR872471-85). The first comprises 1285 nt, and it was related to *Zeya Brook partiti-like virus 1* (GenBank accession: MW389559), an unclassified partitivirus with 64.0% (nt) and 66.2% (aa) of identity. As with the previous virus, this putative genome clustered with *Cryspovirus* and other unclassified partitiviruses, being named *Buriti partiti-like virus 1* (GenBank accession: OQ779235) (Figure 7b). The other sequence (1264 nt) showed association with the genus *Gammapartitivirus* (Figure 7b) and presented identity (61.6% nt and 59.4% aa) with *Atrato Partiti-like virus 2* (GenBank accession: MN661058). This putative virus was tentatively named *Buriti partiti-like virus 2* (GenBank accession: OQ779236).

In addition, an incomplete genome of 720 nt codifying a RdRp domain was obtained from the same *Sa. quasicyaneus* sample (AR872471-85) (Figure 7a). It presents a closer relationship to *Inari deltapartitivirus* (GenBank accession: OP019955), with identities of 70.8% (nt) and 81.2% (aa). Phylogenetic analysis showed that the obtained genomic fragment is related to members of the genus *Deltapartitivirus* (Figure 7c). According to the ICTV, amino acid identity less than or equal to 90% in the RdRp region is one of the criteria for establishing novel viruses in this genus [35]. Therefore, our data suggest a probably novel deltapartitivirus, tentatively named *Buriti deltapartitivirus* (GenBank accession: OQ779234).

#### 3.2.7. Parvoviridae

**Figure 8:**
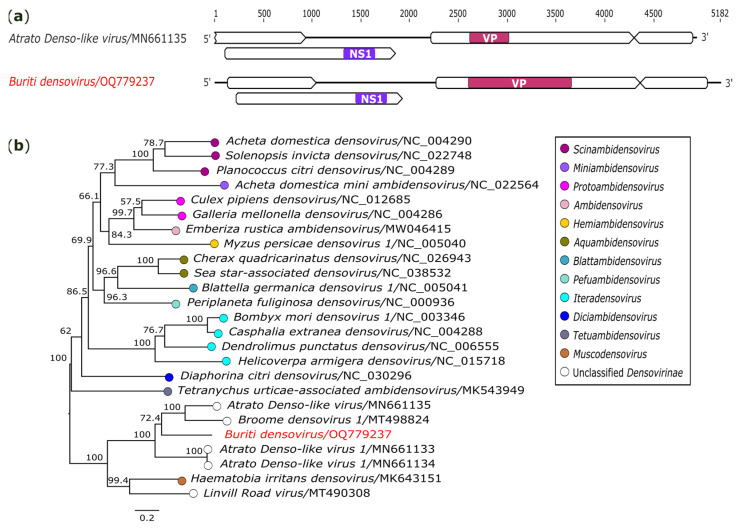
Genomic organization and phylogenetic relationship of *Buriti densovirus*. (**a**) Schematic representation of the obtained sequence compared with members of the subfamily *Densovirinae* (*Parvoviridae*). Domains are displayed as colored boxes, and the sequence size is shown as number of nucleotides. (**b**) Phylogeny tree constructed with nucleotide sequence alignment based on the *NS1* gene. The bar corresponds to the nucleotide diversity along the branch of the tree. The virus identified in this study is indicated in red. VP: capsid viral.

In this investigation, we also report a genome closely related to the subfamily *Densovirinae* (*Parvoviridae*), identified in *Sa. quasicyaneus* (AR872471-85). The recovered sequence has 5182 nt in length and is composed of four ORFs that codify a hypothetical protein (909 nt) and a non-structural protein 1 (NS1) helicase (1704 nt) at the 5′ terminus, and a capsid viral (VP) (2091 nt) and a hypothetical protein (693 nt) at the 3′ terminus (Figure 8a). The complete sequence presented the same genomic architecture and close relationship to the *Atrato Denso-like virus* (GenBank accession: MN661135)*,* an unclassified *Densovirinae.* However, the nucleotide and amino acid alignments of the NS1 helicase domain revealed that these viruses shared 54.2% (nt) and 40.1% (aa) of identity, indicating that we recovered a novel parvovirus that was tentatively named *Buriti densovirus* (GenBank accession: OQ779237).

Phylogenetic analysis based on the NS1 region placed that *Buriti densovirus* in a distinct clade where there are unclassified *Densovirinae* viruses and a representative member of the genus *Muscodensovirus* (Figure 8b).

## 4. Discussion

Metagenomic analysis has provided the exploration of insect virome and contributed to the knowledge of viral diversity present in different ecosystems. In this study, we analyzed the virome of medically important mosquito species that occurred in a transition ecosystem between the Amazon, Cerrado, and Caatinga Brazilian biomes.

The genomes of viruses associated with human and animal diseases were not detected. On the other hand, nine viral-like genomes related to ISVs, four associated with plant viruses, and two undefined putative viruses were identified. ISVs reported here share a common ancestor with viruses classified into the families *Iflaviridae*, *Metaviridae*, *Lispiviridae*, *Rhabdoviridae*, *Xinmoviridae*, and *Parvoviridae*, but all of them are genomically divergent from those previously described, suggesting that they are novel species.

It is important to highlight that the identification of ISVs in mosquitoes demonstrated in this study and their close association with previously described species do not sustain their ability to infect insects. Host specificity can be confirmed through infection studies in different invertebrate cell lines. Therefore, more investigations are necessary to determine if the hypothetical viruses recovered here could replicate in mosquito cells. However, the close relationship of the ISV genomes shown in this investigation with other viruses identified in insect hosts leads to important insights. For example, some of the recovered genomes were closely associated with ISVs involved in the reduction of arboviral infection, as mentioned in the next paragraphs. Thus, we cannot rule out the possibility that the presence of different ISVs inhibits the circulation of arboviruses in the studied region, given that many studies have demonstrated this ISV-arbovirus interaction [5,6,36,37,38].

Members of *Iflaviridae* have a wide host range in the phylum Arthropoda, mostly from the class Insecta [33]. Previously, studies reported that some species could cause damage to many pest insects [39,40,41]. However, iflaviruses have been found in wild-collected mosquitoes, such as *Culex* spp. [42,43,44,45] and *Aedes* spp. [46,47], with no apparent phenotypic abnormalities, indicating that apparently these viruses occur naturally in mosquitoes. In this study, genome-like iflaviruses were obtained from a pool of *Cx.* (*Cux.*) spp. Moreover, other iflavirus species have already been reported in *Ae. aegypti* from the Amazon region of North Brazil [46], showing that more species of this family may be spread in the country.

Additionally, our data showed that two genomes related to the genus *Errantivirus* (*Metaviridae*) co-exist in the studied area. The two genomes were identified in *Sa*. *chloropterus* and *Cx*. *usquatus* and are close to the *Chibugado virus* but deeply divergent between them. Errantiviruses are endogenous retroviruses that integrate into host genomes during replication [48]. This could explain the different genomes found here, given that they were recovered in two mosquito species, or this difference can be associated with a variation acquired over time. The host distribution of errantiviruses in eukaryotes is restricted to insects [49]. In mosquitoes, two other *Errantivirus* species, named *Aedes aegypti To virus 1* and *Aedes aegypti To virus 2*, have already been identified in *Ae*. *aegypti* from Tocantins, North Brazil [50].

Another ISV identified here was classified in the subfamily *Densovirinae* (*Parvoviridae*). Viruses in this subfamily infect insects, decapod crustaceans, and echinoderms and are distributed into six genera [51]. However, the putatively recovered ISV clustered in a distinct clade where there were unclassified *Densovirinae* viruses and a representative member of the genus *Muscodensovirus*. Moreover, it has low amino acid identity with other densoviruses. According to the ICTV, all viruses in a genus should be monophyletic and encode NS1 proteins with amino acid identity above 30% [52]. This indicates that the *Buriti densovirus* and the other closest unclassified viruses could form a novel genus in future revisions. However, the few available sequences related to *Densovirinae* limit inferences.

It is important to highlight that the previously described ISV named *Anopheles gambiae densovirus*, which also belongs to the subfamily *Densovirinae*, negatively affects the *Mayaro virus* (MAYV) infection in *Anopheles gambiae* cells and mosquitoes [38]. MAYV is an important arbovirus circulating in the Amazon basin [53]. Then, our findings can contribute to novel densovirus species that may be used in arbovirus control methods in future investigations.

In this investigation, we also discovered four virus-like contigs related to ISV of the order *Mononegavirales* and belonging to three different families: *Rhabdoviridae*, *Xinmoviridae*, and *Lispiviridae*. The known ISVs, rhabdoviruses, are classified in the genera *Almendravirus* and *Sigmavirus* [54]. However, in our data, the putative ISV clustered with unclassified rhabdoviruses in a clade containing other viruses detected in mosquitoes. Maybe a novel genus of mosquito-specific rhabdoviruses can be created in the next revision. Furthermore, it is important to emphasize that the family *Rhabdoviridae* also includes arboviral species distributed in the genera *Curiovirus*, *Ephemerovirus*, *Hapavirus*, *Ledantevirus*, *Sripuvirus*, *Tibrovirus*, *Vesiculovirus,* and *Caligrhavirus* [54]. Curiously, all the genera of ISVs and arboviruses cited above belong to the subfamily *Alpharhabdovirinae*. This close relationship supports the theory that arboviruses and ISVs may have an ancestral relationship [55,56]. Therefore, reports of novel species belonging to the same arboviral classification may help to elucidate evolutionary questions about mosquito-borne viruses.

In relation to *Xinmoviridae*, this family was established in 2018 for the inclusion of a novel genus, *Anphevirus*, and all species registered until now infect insects [57]. Among xinmoviruses, the species *Aedes anphevirus* has important implications for the surveillance of emerging viral diseases given that it can reduce *Dengue virus* replication in vitro [58]. Unfortunately, this study shows evidence of the occurrence, but the short fragments recovered limit further conclusions about the circulation of *Anphevirus* species in the investigated area. In another previously conducted mosquito investigation from Brazil, another *Xinmoviridae*-related virus, *Aedes anphevirus*, was identified in *Ae*. *aegypti* from the Midwestern region. However, as with our obtained data, the authors recovered a short fragment of 552 nt associated with glycoprotein [59].

As well as *Xinmoviridae*, *Lispiviridae* were established in 2018 for the inclusion of the genus *Arlivirus* and encompass several viruses discovered in invertebrates [57], but only a few pieces of information about them are available. It is important to notice that the growing number of viruses discovered on invertebrates has led the ICTV to realize constant information updates and the creation of new groups to better classify the viruses, as occurred for *Xinmoviridae* and *Lispiviridae*, which emphasizes the importance of metagenomic studies.

Four genomes identified in this study showed a relationship to plant viruses. The finding of these viruses in mosquitoes can be related to the ingested plant sap and nectar required to supply their nutritional needs [15]. These viruses can occupy the invertebrate host intestine temporarily; however, they are nonpathogenic or have limited pathogenicity to their insect vectors [60]. Among the identified viruses, two sequences associated with the families *Solemoviridae* and *Virgaviridae* clustered in distinct clades with unclassified species. Probably, these sequences will also form new genera in the other revisions.

The other plant virus identified in this study was classified in the genus *Deltapartitivirus* (*Partitiviridae*) and comprises a small part of the *RdRp* gene. Despite being a well-conserved region and preferable for determining phylogenetic relationships, this incomplete genome could not reveal a reliable identity shared between these viruses. Additional mosquito collections in the studied area should be performed tentatively to obtain complete sequences to confirm these inferences.

Evidence of other groups of viruses was identified in this investigation. Three virus-like contigs associated with the *capsid* gene cluster with members of genera *Cryspovirus* (which comprises protozoa viruses) and *Gammapartitivirus* (composed by fungi viruses) belong to the family *Partitiviridae* [61]. Unfortunately, capsid is not a preferable gene to establish phylogenetic associations, and these inferences could not reveal a reliable relationship between these viruses. As mentioned above, additional mosquito collections should be performed to obtain the complete genome of these putative viruses. Moreover, the lack of other representative cryspoviruses limits more relationship analysis.

In this study, we identified mutual viruses present in single samples. Viruses belonging to the families *Iflaviridae* (*Inhamum iflavirus*) and *Rhabdoviridae* (*Inhamum rhabdovirus*) were identified in a pool of 13 *Cx.* (*Cux.*) spp. (AR872456), while members of the families *Partitiviridae* (*Pedras virus*, *Buriti partiti-like virus 1*, *Buriti partiti-like virus 2*, and *Buriti deltapartitivirus*) and *Parvoviridae* (*Buriti densovirus*) were identified in a pool composed by 18 *Sa*. *quasicyaneus* (AR872471-85). It Is important to emphasize that it does not suggest the co-existence of viruses given that these samples are composed of a pool of mosquitoes and the recovered genomes could be present in different individuals.

Perhaps a longer period of mosquito sampling and exploration of other transition areas would recover a greater diversity of viruses in the studied region. For this, we suggest analyzing the amplitude of time and cover area in future investigations. In addition, this research highlights the application of NGS in identifying different groups of viruses and unknown species that would not have been possible using conventional methods. Nowadays, ICTV recognizes that genomes assembled from metagenomic data contain sufficient information to be incorporated into the official classification scheme, given that this procedure is indispensable for the comprehensive characterization of the global virome [62]. Here we show evidence of genetic sequences associated with viruses, but we also suggest that more studies addressing the biological properties of these putative viruses could be performed to complement these results.

## 5. Conclusions

This study revealed that the virome of mosquitoes collected from transition areas in the north and Northeast of Brazil was diverse, with viral-like genomes classified into nine taxonomic families and associated with insect-specific and plant viruses. Notably, viruses associated with the invertebrate host were prevalent, and all of them are novel species, which could be of great importance to arbovirus control methods in future investigations. Furthermore, these findings expand our knowledge about the virosphere.

## Figures and Tables

**Table 1:** Descriptions of the mosquito samples used for metagenomic analysis and generated data.

Sample Name	Species	Number of Individuals	Locations	Collection Date	Raw Reads	Reads after Fastp Treatment	Reads after SortMeRNA Treatment
AR872456	*Culex (Culex)* spp. ^1^	13	CX ^2^	19 October 2021	35,747,762	33,641,832	17,581,122
AR872459	*Aedes serratus*	4	CX ^2^	20 October 2021	23,234,440	21,562,940	14,833,379
AR872460	*Aedes albopictus*	1	CX ^2^	21 October 2021	38,297,936	34,832,610	14,935,751
AR872461	*Aedes serratus*	20	CX ^2^	20 October 2021	24,493,722	23,393,402	2,480,108
AR872465	*Sabethes chloropterus*	1	CX ^2^	21 October 2021	22,534,910	21,033,654	8,606,130
AR872468-76	*Aedes albopictus*	6	TM ^3^	25 October 2021	25,453,046	23,883,884	11,137,227
AR872471-85	*Sabethes quasicyaneus*	18	TM ^3^	25 October 2021	27,797,262	26,406,522	26,216,665
AR872474	*Aedes fluviatilis*	2	TM ^3^	25 October 2021	16,882,686	15,932,038	4,184,885
AR872475	*Sabethes chloropterus*	2	SJS ^4^	25 October 2021	40,165,978	37,486,668	34,960,981
AR872486	*Sabethes chloropterus*	2	TM ^3^	25 October 2021	22,567,056	20,857,106	4,593,808
AR872487	*Sabethes glaucodaemon*	2	TM ^3^	25 October 2021	38,840,018	36,253,540	14,174,187
AR872498	*Culex usquatus*	1	TM ^3^	25 October 2021	65,179,918	61,690,026	29,604,510
AR872499	*Culex (Melanoconion)* spp. ^1^	4	TM ^3^	25 October 2021	52,819,748	49,845,674	31,194,633
AR872505-09	*Aedes scapularis*	3	SJS ^4^	26 October 2021	31,780,386	29,756,330	14,765,661
AR872508	*Sabethes quasicyaneus*	1	SJS ^4^	26 October 2021	7,430,430	7,020,402	1,460,524
AR872510	*Aedes serratus*	9	SJS ^4^	26 October 2021	31,152,824	29,451,912	6,142,182
AR872511	*Aedes albopictus*	2	SJS ^4^	26 October 2021	39,201,792	36,788,368	9,662,953
AR872515	*Haemagogus janthinomys*	1	SJS ^4^	27 October 2021	6,970,298	6,527,944	3,606,142
AR872521	*Aedes scapularis*	1	TM ^3^	26 October 2021	27,952,604	26,417,466	6,581,716
AR872524	*Aedes scapularis*	1	SJS ^4^	27 October 2021	29,945,114	27,968,266	17,607,240
Total	*-*	94	-	-	608,447,930	570,750,584	274,329,804

^1^ Species; ^2^ Caxias (S 04°55′12.7″ W 043°27′37.0″); ^3^ Proximity of Timon (S 04°58′50.4″ W 043°07′51.2″); ^4^ São João do Sóter (S 04°59′12.7″ W 043°35′59.1″).

**Table 2:** Putative viral sequences obtained in mosquitoes from a transition ecosystem of north-northeast Brazil and their best hits compared with the NCBI non-redundant (nr) database using BlastX.

Molecule Type	Virus Name (GenBank Accession)	Sample/Host	Length (nt)	Mean Coverage	Classification	Closest Virus (GenBank Accession)	BlastX			
							Region	Amino Acid Identity (%)	QC ^1^ (%)	E-Value
+ssRNA	*Inhamum iflavirus* (OP918263)	AR872456/*Cx*. (*Cux*.) spp.	9020	16x	*Iflaviridae*	*Culex Iflavi-like virus 3* (MW434116)	Pol ^2^	58.00	99	0.0
	*Inhamum errantivirus* (OQ779233)	AR872465/*Sa*. *chloropterus*	5823	34.1x	*Metaviridae*	*Chibugado virus* (MN661043)	Pol ^2^	64.31	99	0.0
	*Buriti errantivirus* (OQ779240)	AR872498/*Cx*. *Usquatus*	5014	16.5x	*Metaviridae*	*Chibugado virus* (MN661043)	Pol ^2^	63.42	98	0.0
	*Atrato Sobemo-like virus 1* (OQ779232)	AR872461/*Ae. serratus*	2726	547.7x	*Solemoviridae*	*Atrato Sobemo-like virus 1* (MN661087)	RdRp ^3^	97.37	99	0.0
	*Atrato Sobemo-like virus 1* (OQ779242)	AR872510/*Ae. serratus*	1451	1861.8x	*Solemoviridae*	*Atrato Sobemo-like virus 1* (MN661087)	RdRp ^3^	98.76	99	0.0
	*Buriti virga-like virus* (OQ779238)	AR872486/*Sb. Chloropterus*	8757	1878.4x	*Virgaviridae*	*Atrato Virga-like virus 2* (MN661104/MN661105)	Pol ^2^	60.86	99	0.0
−ssRNA	*Pedras lispivirus* (OQ779241)	AR872508/*Sa. quasicyaneus*	13,424	237.8x	*Lispiviridae*	*Canya virus* (MW434766)	RdRp ^3^	36.24	99	0.0
	*Inhamum rhabdovirus* (OQ779231)	AR872456/*Cx.* (*Cux.*) spp.	6397	11.5x	*Rhabdoviridae*	*Stang virus* (MW434775)	RdRp ^3^	77.36	99	0.0
	*Buriti anphevirus* (OQ779239)	AR872487/*Sa. glaucodaemon*	1139	1441.1x	*Xinmoviridae*	*Enontekio anphevirus 2*/ON955256	RdRp ^3^	53.44	99	2E-144
	*Pedras xinmovirus* (OQ779244)	AR872521/*Ae. scapularis*	787	3.3x	*Xinmoviridae*	*Guadeloupe mosquito mononega-like virus*/MN053735	N ^4^	87.20	100	2E-132
dsRNA	*Pedras partitivirus* (OQ779243)	AR872511/*Ae. albopictus*	1364	5.5x	*Partitiviridae*	*Verdadero virus* (MT742175)	Capsid	80.81	98	0.0
	*Buriti partiti-like virus 1* (OQ779235)	AR872471-85/*Sa. quasicyaneus*	1285	358.6x	*Partitiviridae*	*Zeya Brook partiti-like virus 1* (MW389559)	Capsid	66.16	99	2E-87
	*Buriti partiti-like virus 2* (OQ779236)	AR872471-85/*Sa. quasicyaneus*	1264	4.6x	*Partitiviridae*	*Atrato Partiti-like virus 2* (MN661058)	Capsid	58.57	93	3E-69
	*Buriti deltapartitivirus* (OQ779234)	AR872471-85/*Sa. quasicyaneus*	720	3.6x	*Partitiviridae*	*Inari deltapartitivirus* (OP019955)	RdRp ^3^	84.69	99	9E-127
ssDNA	*Buriti densovirus* (OQ779237)	AR872471-85/*Sa. quasicyaneus*	5182	1684.9x	*Parvoviridae*	*Atrato Denso-like virus* (MN661135)	NS1 ^5^	42.42	91	7E-144

^1^ Query cover; ^2^ Polyproteins; ^3^ RNA-dependent RNA polymerase; ^4^ Nucleoproteins; ^5^ Non-Structural Proteins 1.

## Data Availability

The raw sequence reads generated in this study are available at the NCBI Sequence Read Archive (SRA) database under BioProject PRJNA947063 and BioSamples SAMN33837849 to SAMN33837868. All virus contigs generated in this study have been deposited in GenBank under accession numbers: OP918263, and OQ779231 to OQ779244.
